# Students in London and Paris

**Published:** 1860-05

**Authors:** 


MEDICAL STUDENTS IN LONDON AND PARIS.—The number of medical students in London during the past winter was 1063, being 42 more than the previous session. In Paris the number was 988, including 66 for the grade of Officer of Health; of this number 304 were new inscriptions. In 1858, the total number was 1055, embracing 251 new matriculates. It will thus be seen that a much larger number of students was assembled in this city during the last winter than either in the British or French metropolis. The number of foreign students in Paris this year is 48; last year it was only 34.

